# Mediastinal thymoma in a patient with previous rectal and breast cancers: A report of a case with multiple primary cancers and review of literature

**DOI:** 10.1002/ccr3.5987

**Published:** 2022-06-21

**Authors:** Parviz Mardani, Mohammadmehdi Fallahi, Hooman Kamran, Reza Shahriarirad, Mohammad Hossein Anbardar, Neda Soleimani

**Affiliations:** ^1^ Thoracic and Vascular Surgery Research Center Shiraz University of Medical Science Shiraz Iran; ^2^ Students Research Committee, School of Medicine Shiraz University of Medical Sciences Shiraz Iran; ^3^ Department of Pathology Abu Ali Sina Hospital Shiraz Medical School Shiraz University of Medical Sciences Shiraz Iran

**Keywords:** breast neoplasms, metachronous neoplasms, multiple primary cancers, multiple primary neoplasms, second primary neoplasms, thymus neoplasms

## Abstract

A 42‐year‐old female patient with intellectual disability was presented to us as a newly diagnosed case of thymoma. She was identified as a case of multiple primary cancers, including adenocarcinoma of the rectum, carcinoma of the breast, and mediastinal thymoma, in a 15‐year period, who underwent chemotherapy, radiotherapy, and surgical resection.

## Graphical abstract

Chest computed tomography scan of a 42‐year‐old female patient case of multiple primary cancer (previous rectal and breast cancer) diagnosed with a mediastinal thymoma; A) coronal view, B) sagittal view, and C) axial view.

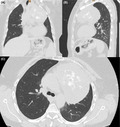

## 1 INTRODUCTION

Multiple primary cancers (MPC) or multiple primary malignant neoplasms (MPN) is defined as the occurrence of at least two malignant neoplasms in an individual regardless of the time and organ of detection with no metastatic origin.^1^ Metachronous multiple primary cancers occur in at least 6‐month period of time. Herein, we present a case of a patient with metachronous neoplasms of the rectum, breast, and mediastinum.

## 2 CASE PRESENTATION

A 42‐year‐old female patient with intellectual disability and a previous history of treated breast and rectal cancer was referred to our center for the resection of a mediastinal mass, diagnosed as thymoma. She had stable vital signs in the physical examination.

Fifteen years prior to the admission, she underwent rectosigmoid resection, and colostomy insertion was performed regarding the treatment of rectal cancer. The diagnosis of rectal cancer was adenocarcinoma (infiltrative well‐differentiated). Also, 4 years prior to the admission, the patient underwent partial right mastectomy with 21 sessions of chemotherapy and 30 sessions of radiotherapy session after the surgery of her breast cancer. The breast mass was reported as invasive mucinous carcinoma. Besides, for 8 months before the admission, she was taking rivaroxaban due to the right‐hand deep vein thrombosis.

A previous computed tomography (CT) scan had shown an enlarged left lobe of the thyroid and a large centrally calcified anterior mediastinal mass, pointed to the left side, measuring in dimension about 78 × 52 mm at the level of the aortic arch. Fine needle aspiration of the left thyroid lobe reported benign follicular nodule (adenomatous goiter with focal lymphocytic thyroiditis), and the tru‐cut biopsy of the mediastinal mass showed epithelial neoplasm with foci vagus glands formation.

A year later, she had taken another chest CT, which showed a mass containing calcification with a size of approximately 124 × 113 × 80 mm in the anterior mediastinum. The mass was extended to the thoracic inlet with heterogeneous enhancement after contrast injection. Another tru‐cut biopsy was taken. According to the immunohistochemical study and morphology, the tumor was reported to be a thymoma type A. The immunohistochemical study result was positive for LCA (rarely positive), CK, EMA (weak scattered positive), CD5 (rare scattered positive), and HMWCK (rare scattered positive) and was negative for GATA3, PAX‐8, CDX2, CK7, CK20, and desmin. Therefore, the patient was referred to our center for tumor resection.

Thyroid evaluation had been performed before operation, revealing nothing in favor of hypothyroidism or hyperthyroidism. So, performing the surgery was safe and unimpeded.

**FIGURE 1:**
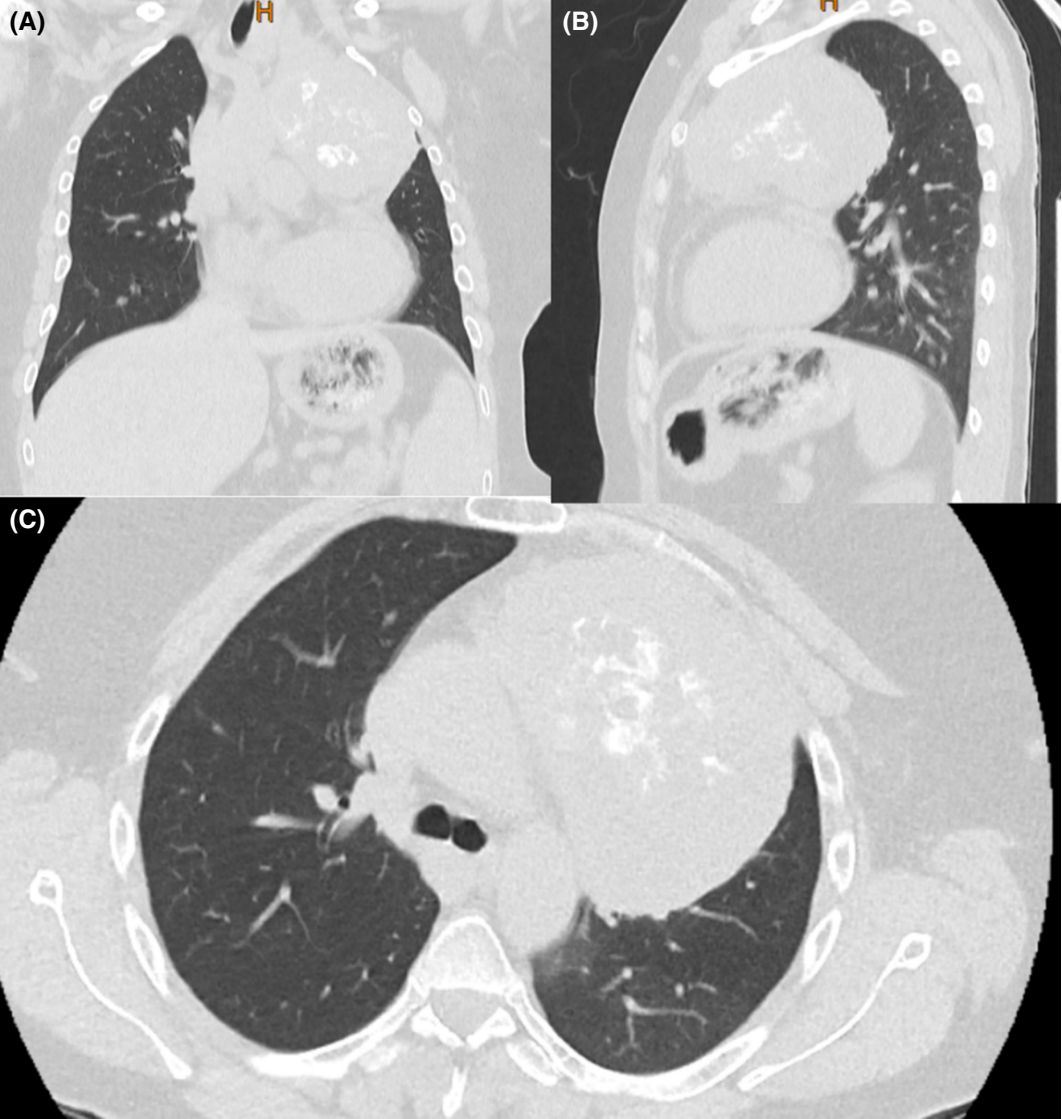
Chest computed tomography scan of a 42‐year‐old patient with a mediastinal thymoma; (A) coronal view, (B) sagittal view, and (C) axial view

We performed a CT scan (Figure 1) and operated on the patient. After general anesthesia in the supine position, a complete sternotomy was done. Exploration showed a very large anterior mediastinal mass with an extension to the apex of the left chest cavity and inferior of the neck with an encasement of innominate vein and adhesion to great vessels of the aortic arch. The mediastinal mass was removed from the tissues and organs to which it was attached and was resected completely. Besides, due to the large nodule of the left lobe of the thyroid, thyroidectomy was done. Also, a thymectomy was performed. The operation ended successfully with bilateral mediastinal pleurectomy and bilateral chest tube insertion. The resected mass was sent for pathological evaluation.

**FIGURE 2:**
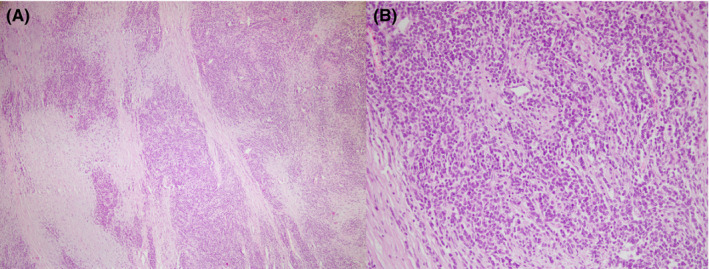
Microscopic section of the mediastinal mass; (A) microscopic section of mediastinal mass shows low power view of lobulated architecture with intersecting bands (Hematoxylin and Eosin, ×40); (B) microscopic section shows cellular polygonal epithelial cells with rare lymphocytes (Hematoxylin and Eosin, ×200)

**FIGURE 3:**
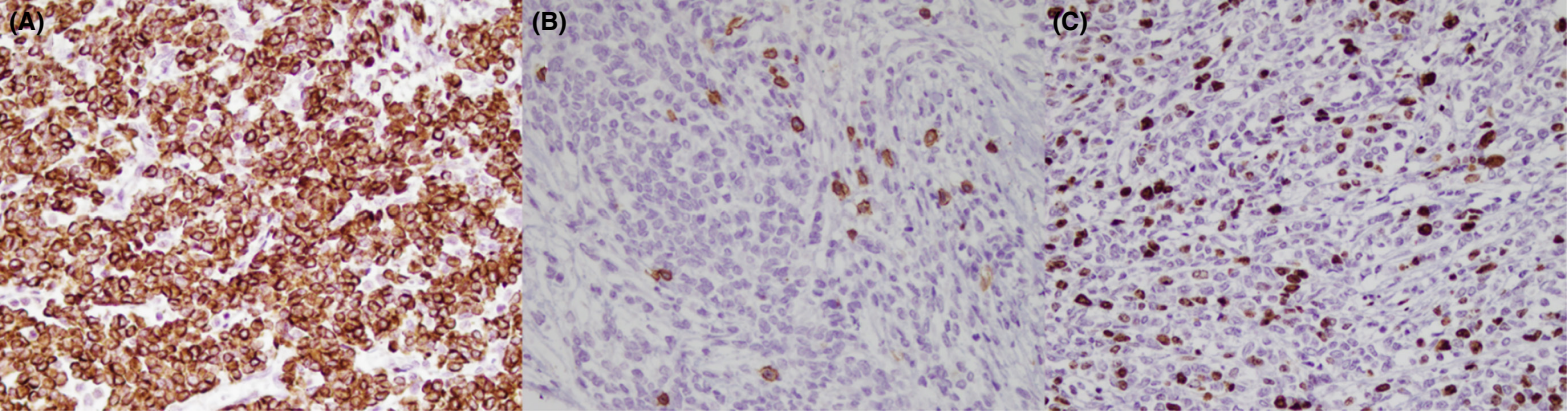
Pathological evaluation of the mediastinal mass; (A) immunoreactivity of epithelial cells for cytokeratin (×400); (B) immunoreactivity of lymphocytes for CD 5 (×400); (C) Ki‐67 proliferative index (×400)

Gross examination showed a large semi‐ovoid gray well‐defined rubbery mass measuring 12 × 10 × 7 cm attached to the thymus. Cut sections showed a homogenous creamy surface with foci of hemorrhage and ossification. Histopathologic examination revealed lobulated architecture with cellular lobules and intersecting fibrous bands (Figure 2A). Neoplastic epithelial cells were polygonal with moderate atypia and mitosis (Figure 2B). No necrosis was identified. There were rare lymphocytes. The capsular invasion was seen; however, no lymphovascular invasion was identified. Immunohistochemical study of epithelial cells showed immunoreactivity for cytokeratin (Figure 3A) but no immunoreactivity for CD 5, CD117, p63, TTF‐1, chromogranin, synaptophysin, CK 5/6, and monoclonal PAX‐8. Rare lymphocytes were immunoreactive for CD 45 and CD 5 (Figure 3B). Ki‐67 proliferation index was about 30% (Figure 3C). The diagnosis of thymoma (WHO) type B3 was confirmed according to the histopathological and immunohistochemical study.

## 3 DISCUSSION

During the past decades, developments in the diagnosis and treatment of patients with cancer have improved life expectancy in cancer patients and introduced new findings in survivors of this disease.^2^ The incidence of subsequent primary cancer is higher in cancer survivors than in the normal population.^3^, ^4^ The patient in our presentation fills the Warren and Gates criteria for second primary malignancy (SPM) and ICD‐0 (third edition)^1^, ^5^ for metachronous multiple malignancies as follows: (1) Histological confirmation of infiltrative well‐differentiated adenocarcinoma of the rectum, invasive mucinous carcinoma of the breast, and thymoma type B3; all of which were separated by normal mucosa and not related to one another by histological origin, (2) the occurrence of three tumors was chronologically separated by at least 2 years. According to previous studies, breast cancer is one of the most common SPMs following colorectal cancer,^3^, ^6^ which was seen in our case.

Souadjian et al. published one of the first papers on the relation between thymoma and occurrence of other primary cancers, in which the prevalence of other non‐thymic malignant lesions was 21% in thymoma cases in contrast with 8% in parathyroid cancer cases, which was presented as the control group.^7^ Also, there are few cases in literature for MPCs, including thymoma; Šitić et al. presented an almost similar case of a patient with breast cancer, rectal adenocarcinoma, lung carcinoma, and thymoma^8^; Tanimura et al. reported a series of 97 cases presented with thymoma, in 7 of which another malignant lesion in other organs was also presented (1 case of breast and 1 case of colon)^9^; Tehrani et al. reported 2 cases of MPCs including thymoma^10^; Athanasiou et al. reported a case of MPCs, including thymoma and breast cancer^11^; and Welsh et al. presented 136 cases of thymoma, in which in 38 cases, a second and even a third neoplasm also occurred (12 cases of colorectal carcinoma and 4 cases of breast carcinoma)^12^ (Table 1).

**TABLE 1:** Literature review of multiple primary cancers (MPC), including thymoma

Authors	Year	Cases	Other malignancies	Comments
Welsh et al.	2000	38 cases of MPC in 136 cases of thymoma (28%)	12 cases of colorectal carcinoma (8% of all cases) 4 cases of breast carcinoma (2% of all cases)	Suggests that radiation therapy for thymoma has no meaningful effect on the prevalence of subsequent malignancies
Tanimura et al.	2002	Seven cases of MPC in 97 cases of thymoma (7%)	1 case of breast cancer (1% of all cases) 1 case of colon cancer (1% of all cases)	–
Šitić et al.	2008	One case of MPC	Breast cancer, rectal adenocarcinoma, lung carcinoma, thymoma	–
Tehrani et al.	2010	Two cases of MPC	1 case of melanoma, multiple myeloma, liposarcoma, thymoma and basal cell carcinoma 1 case of colorectal carcinoma and thymoma	Thymoma‐associated malignancies may herald a hereditary cancer syndrome independent of TP53 and chromosomal mutations
Athanasiou et al.	2016	One case of MPC	Breast carcinoma and thymoma	–

Causes of MPC have not been systematically studied; yet, Motuzyuk et al. suggest the following as probable causes: (1) Endogenous factors, such as abnormal embryonic development, immunity‐related diseases, and endocrine diseases affecting sensitivity to carcinogens, (2) environmental and lifestyle exposures, including long‐term effects of radiation and industrial pollution, (3) genetic determinants, such as BRCA 1/2 genes, and (4) iatrogenic effect, especially radiation therapy and drug therapy.^13^

Genetic predisposition plays an important role in presenting cancer in individuals,^14^ and there are known inherited cancer syndromes that are caused by single or multiple gene mutations.^15^, ^16^ A case of thymoma was reported by Principe et al., which was accompanied by deleterious BRCA2 mutation, a gene mostly seen in breast cancer but proven unrelated to colorectal cancer.^17^ Also, another case of thymoma was reported by Tampellini et al., which was accompanied by Lynch syndrome.^18^ In another case series, three patients with a family history of upper aero‐digestive tract squamous carcinoma developed MPC, suggesting an inherited predisposition.^19^ Kotnis et al reported in distinct ethnic groups, risk for different cancers could be modulated by interaction between different exogenous and endogenous carcinogens and genetic variants.^20^ Increasing biological and genetical understanding of MPC could assist in management planning and early diagnosis and preventing of carcinogenetic risk factors.

Intellectual disability, as the sole past medical condition of our patient, has been investigated as an associated condition with cancer in a cohort study by Liu et al., in which they showed that the prevalence of any type of cancer was higher in patients with intellectual disability that could not be explained by heritability. Additionally, they reported statistically significant associations for cancers of the digestive system and intellectual disability.^21^ Proposed mechanisms for this association include the role of multiple‐system congenital anomalies^22^ and extrinsic factors, such as lack of fiber intake and low physical exercise.^23^

## 4 CONCLUSION

MPC is a rare condition in the literature, and only a few studies have reported and described it, but as the cancer survivor population continues to grow, the need for an evidence‐based protocol for evaluation and screening for it also arises. We also recommend a similar strategy for patients with an intellectual disability, considering the poor emphasis on a healthy lifestyle and possible lack of expression of signs and symptoms related to other malignancies.

## AUTHOR CONTRIBUTIONS

M.A. and N.S. made the histopathological diagnosis of the case, while P.M. carried out the therapeutic measures. M.F. and H.K. collected the data and drafted the manuscript. H.K. and R.S. revised and proofread the manuscript. All authors read and approved the final version of the manuscript.

## CONFLICT OF INTEREST

The authors declare that they have no competing interests.

## ETHICS APPROVAL

The present study was approved by the Medical Ethics Committee of Shiraz University of Medical Sciences.

## CONSENT

Written informed consent was obtained from the patient for the publication of this case report and any accompanying images. A copy of the written consent is available for review by the Editor of this journal.

## Data Availability

All data regarding this case report has been reported in the manuscript. Please contact the corresponding author in case of requiring any further information.
